# 2017–2018 Assisted Reproduction Cost Analysis Performance Indexes: Lombardy County Case Study

**DOI:** 10.3389/frph.2021.693715

**Published:** 2021-08-12

**Authors:** Paolo Emanuele Levi-Setti, Andrea Busnelli, Annalisa Bodina, Roberto De Luca, Giulia Scaravelli

**Affiliations:** ^1^Department of Biomedical Sciences, Humanitas University, Milan, Italy; ^2^Division of Gynecology and Reproductive Medicine, Department of Gynaecology, Fertility Center, IRCCS Humanitas Research Hospital, Milan, Italy; ^3^Direzione Generale Welfare, Regione Lombardia, Milan, Italy; ^4^Assisted Reproduction Techniques Italian National Register, National Centre for Diseases, Prevention and Health Promotion, National Health Institute, Rome, Italy

**Keywords:** ART performance, cumulative delivery rate, cost-analysis, public reimbursement, freeze importance

## Abstract

**Objective:** The aim of the present study was to analyze the IVF success rates and the economic cost per delivery in all the public funded IVF Units in Lombardy in the 2017–2018 period and to assess any significant difference in ART outcomes among the enrolled centers.

**Methods:** Analysis of costs for the 2017 and 2018 fresh transfer delivery rate (DR) and Cumulative delivery rate (CDR) considering both fresh and frozen cycles were extracted from the ART Italian Registry on oocytes retrievals, fresh and frozen embryos and oocytes embryo transfer performed in 22 Lombardy IVF Units.

**Results:** In 2017, 29,718 procedures were performed, resulting in 4,543 pregnancies and 3,253 deliveries. In 2018, there were 29,708 procedures, 4,665 pregnancies and 3,348 deliveries. Pregnancies lost to follow up were 5.0% with a (range of 0–67.68%) in 2017 and 3.4% (range of 0–45.1%) in 2018. The cost reimbursement for the cycles were €2,232 ($2,611) for oocyte retrieval and €2,194 ($2,567) for embryo transfer, excluding ovarian stimulation therapy and luteal phase support. 19.33 (5.80). The DR was 13.23 ± 5.69% (range 2.86–29.11%) in 2017 and 19.33 ± 5.80% in 2018 (range 11.82–34.98 %) and the CDR was 19.86 ± 9.38% (range 4.43–37.88%) in 2017 and 21.32 ± 8.84% (range 4.24–37.11%). The mean multiple pregnancy delivery rate (MDR) was 11.08 ± 5.55% (range 0.00–22.73%) in 2017 and 10.41 ± 4.99% (range 1.33–22.22%) in 2018. The mean CDR cost in euros was 26,227 ± 14,737 in 2017 and 25,018 ± 16,039 in 2018. The mean CDR cost among centers was 12,480 to 76,725 in 2017 and 12,973 to 86,203 in 2018.

**Conclusions:** Our findings show impressive differences in the DR and CDR among centers and the importance of cryopreservation in patients' safety and economic cost reduction suggesting the formulation of specific KPI's (Key performance indexes) and minimal performance indexes (PI) as a basis for the allocation of public or insurance resources. In particular, the reduction of multiple pregnancy rates costs, may lead to a more widespread use of ART even in lower resources countries.

## Key Message

2017–2018 all Italian, Lombardy County public funded IVF centers cost analysis performance indexes show an impressive variability in cumulative delivery rates, not justified by population treated variables.

## Introduction

The aim of the present study was to analyze the IVF success rates and the economic cost per delivery in all the public funded IVF Units in Lombardy county in the 2017–2018 period and to assess any significant difference in ART outcomes among the enrolled centers. We also investigated the impact of multiple pregnancy rate (MPR) and lost to follow up pregnancies.

Reimbursement of ART (Assisted Reproduction Techniques) treatments by government-funded programs and third-party payers are not uniform but rather variable. In the latest (2019) global survey of ART practices and policies undertaken by the International Federation of Fertility Societies, only 40 (47%) of the 85 countries who submitted data on the extent of insurance coverage, reported any type of financial support for ART treatment (1). Such differences influence greatly the number of couples able to access treatment (2–4).

Live birth rates per individual embryo transfer are increased when more than one embryo is transferred, therefore, when patients must pay for treatments as out-of-pocket expenses, or when only a small number of treatment cycles are reimbursed, there is an economic incentive to achieve pregnancy, risking higher multiple pregnancy rates (5, 6). A number of studies have shown that the costs of government funding for ART treatments can be theoretically off-set by savings in the health care costs of caring for ART-conceived multiple- birth infants (7).

Comparing results between continents, countries and even regions remains extremely complicated for a variety of reasons, such as huge differences in the cost of treatment and associated differences in access to care, along with differences in the characteristics of the patients being treated (8). Theoretically, national registry data should reflect a more homogeneous background, but data show that the IVF success rate still varies between the different public-funded IVF centers (9). Lintsen et al. investigated whether differences in pregnancy chances among IVF centers remained after controlling for the type of patients treated, reporting differences in 1-year ongoing pregnancy rates between IVF centers even after adjustment for sampling variation and patient mix. According to the authors, future investigations in order to analyze this issue should change perspective and look beyond patients-related factors (9). The importance of big data to support randomized trials and meta-analyses has been recently reinforced (10, 11).

In Italy gonadotrophins are supported by National regulation for all public and private practice providing ART cycles as well as for intrauterine inseminations and ovulation induction, up to the age of 45 years. ART cycles, however, are supported with very different reimbursement rates among the 20 Italian counties. The lack of a national regulation of ART, despite legal discussions and proposals to consider ART as an essential medical service requiring assistance, may explain the different county regulations and public vs. private ART funding opportunities. For example, ART cycles performed in 2017 revealed a mean of 32% (ranging from 86% in Sicily to 0% in Sardinia) of cycles performed in private practice arrangements. Lombardy county alone, a region in northern Italy with more than 10 million inhabitants. In its 22 IVF centers, in 2018 were performed 29.8% of the ovulation inductions (13,807/32,580), 31% of fresh and frozen procedures (30,049/66,974) of the entire country.

Interestingly, 99.0% of the procedures performed in Lombardy were funded by the public health system, although a small number (<1% were private practice cycles). All the IVF Centers are in hospitals with a gynecological division, often with an Ob-Gyn and emergency department. For all these reason, Lombardy constitutes an ideal case study to compare data on reimbursement and cost per treatment even with the bias of data being reported in aggregate to the National ART Register (12).

## Materials and Methods

Data on the number of couples, ovulation induction cycles, fresh and frozen (embryos and oocytes) procedures and outcome of all ART performed in all the 22 Lombardy county public funded IVF Units were extracted from the Assisted Reproductive Technology (ART) Italian National Register (National Center for Diseases, Prevention and Health Promotion, National Health Institute, Rome, Italy) for the years 2017 and 2018. Treatment cycles using donor gametes were excluded.

For each ART Center we calculated: the delivery rate per retrieval, the cumulative delivery rate (CDR) per retrieval (13); the multiple pregnancy rate (MPR); the mean live birth cost (weighted for the number of live births achieved by the single IVF Units); and the lost to follow up rate.

CDR is an estimate (not a true rate, as the data set presented here is cross-sectional) of a cumulative rate, calculated from the fresh ET and those carried out after thawing. The data are presented based on the sum of the fresh, FER (Frozen Embryo Replacement) and FO (Frozen Oocytes) deliveries and the number of aspirations of the same year as the denominator. This parameter was chosen according to the European Register (3) and delivery was defined as the expulsion or extraction of one or more fetuses from the mother after 24 completed weeks of gestational age, modified from International Committee for Monitoring Assisted Reproductive Technology (ICMART) and the World Health Organization (WHO) revised glossary of ART terminology, 2009 (14).

Data about COH (Controlled Ovarian Hyperstimulation), luteal phase support and endometrial preparation protocols adopted by each IVF Unit were not available. Costs for pharmacological compounds cannot thus be included in the analysis. Costs for the IVF cycles were retrieved from the local Diagnosis-Related Group (DRG) reimbursements (http://www.e-drg.it/drg24/Codici.htm). Specifically, they were as follows: €225 (US dollar equivalent $263) for cycle preparation and monitoring (this cost item is reimbursed only for canceled COH cycles), €2,232 ($2,611) for oocyte retrieval and €2,194 ($2,567) for embryo transfer. Pregnancy and delivery costs were not included in the cost analysis. For each included Unit, the total cost for the IVF performed procedures was divided by the number of deliveries. Data analysis was performed using the Statistics Package for Social Sciences (SPSS 18). Data was described as mean ± SD, median (Interquartile range: IQR) or number (%) and were compared using Student's *t*-test or the “N-1” chi-squared test as appropriate. *P*-values below 0.05 were considered statistically significant.

The number of couples treated and procedures performed by each Center was not included to assure anonymity (15). However, a relation between performing more than 500 cycles was found with oocyte and embryo cryopreservation higher performance (16) and was already published in 2016.

## Results

In 2017 and 2018, Lombardy region counted 22 public funded IVF Clinics which treated 11,577 and 11,221 infertile couples, respectively. Characteristics and outcomes of IVF cycles are reported in Table 1. The mean delivery rate for fresh retrievals (Table 2) was 13.23% ± 5.69 SD in 2017 (range 2.86–29.11%) and 19.33 ± 5.80% SD (range 11.82–34.98%) in 2018 with a mean improvement of 6.10% ± 4.45 SD (range −1.66 to 15.13%).

**Table 1:** Characteristics and outcomes of IVF cycles performed in public funded IVF Units in Lombrdy county in 2017 and 2018.

**Characteristic**	**2017**	**2018**
Public funded IVF Units (*N*)	22	22
Nr of COH cycles	15,177	14,968
Nr of oocyte retrievals	13,887	13,658
Nr of fresh ETs	10,190	9,770
Nr of FETs	5,342	5,996
Nr of FO cycles	299	284
Rate of women younger than 35	28 ± 8%	28% ± 5
Mean nr of fresh and frozen procedure per IVF Unit	1.35 ± 1.25	1.35 ± 1.28
Nr of pregnancies achieved	4,543	4,665
Nr of deliveries	3,253	3,348
Nr of live births	3,590	3,665
Pregnancies lost to follow-up	227 (5%)	160 (3%)

*IVF, in vitro fertilization; COH, controlled ovarian hyperstimulation; ET, embryo transfer; FET, frozen embryo transfer; FO, frozen oocytes*.

**Table 2:** Delivery rate % for fresh transfer in the 22 IVF centers in the period 2017–2018.

	**Delivery rate fresh cycle** **×** **retrieval %**		
**ART CENTER**	**2017**	**2018**	**DELTA**
C1	13.55	14.49	0.94
C2	21.01	19.34	−1.66
C3	17.91	23.83	5.92
C4	13.27	15.57	2.30
C5	8.55	13.04	4.48
C6	14.53	23.42	8.89
C7	6.94	22.08	15.13
C8	8.82	13.08	4.25
C9	17.18	21.72	4.54
C10	29.11	34.98	5.87
C11	2.86	11.82	8.95
C12	15.84	30.88	15.03
C13	13.53	20.93	7.40
C14	16.30	17.83	1.53
C15	9.47	13.49	4.02
C16	7.69	21.41	13.71
C17	10.87	19.08	8.21
C18	9.42	14.48	5.06
C19	18.99	23.49	4.51
C20	14.02	15.99	1.97
C21	12.96	16.41	3.44
C22	8.26	17.86	9.60
**Mean** **±** **SD**	**13.23 (5.69)**	**19.33 (5.80)**	**6.10 (4.45)**
**Minimum**	**2.86**	**11.82**	**−1.66**
**Maximum**	**29.11**	**34.98**	**15.13**

The CDR per oocyte retrieval was 19.86 ± 9.38% (4.43–37.88%) in 2017 and 21.32% ± 8.84 (4.24–37.11%) in 2018 (Table 3). Only a small overall average improvement was observed between mean 2017–2018 performance (1.46 ± 4.03%), with no real differences in best and poor performance centers (Table 4).

**Table 3:** Cumulative delivery rate (CDR) in all 22 IVF centers in the period 2017–2018.

	**Cumulative Delivery rate %**		
**ART CENTER**	**2017**	**2018**	**DELTA**
C1	25.81	26.57	0.76
C2	28.06	24.35	−3.71
C3	18.54	17.50	−1.04
C4	19.44	24.85	5.41
C5	26.95	30.69	3.75
C6	32.40	36.08	3.67
C7	11.11	19.48	8.37
C8	8.82	6.92	−1.90
C9	24.95	26.71	1.76
C10	37.67	30.80	−6.87
C11	4.43	4.24	−0.18
C12	16.83	25.81	8.97
C13	13.91	15.12	1.21
C14	25.30	21.02	−4.29
C15	20.33	21.63	1.29
C16	8.31	11.01	2.70
C17	16.21	21.97	5.76
C18	11.23	12.46	1.23
C19	37.88	37.11	−0.77
C20	20.56	18.33	−2.23
C21	19.91	26.17	6.26
C22	8.26	10.16	1.90
**Mean** **±** **SD**	**19.86 (9.38)**	**21.32 (8.84)**	**1.46 (4.43)**
**Minimum**	**4.43**	**4.24**	**−6.87**
**Maximum**	**37.88**	**37.11**	**8.97**

**Table 4:** Multiple delivery rate (MDR) in all 22 IVF centers in the period 2017–2018.

	**Multiple Delivery rate %**		
**ART CENTER**	**2017**	**2018**	**DELTA**
C1	3.78	1.33	−2.45
C2	14.10	10.76	−3.34
C3	15.60	12.78	−2.82
C4	10.00	2.35	−7.65
C5	3.67	6.13	2.47
C6	0.00	10.94	10.94
C7	13.51	13.46	−0.05
C8	14.29	18.18	3.90
C9	8.83	10.48	1.65
C10	14.06	10.99	−3.07
C11	15.00	12.50	−2.50
C12	22.73	22.22	−0.51
C13	15.91	15.22	−0.69
C14	5.45	4.81	−0.65
C15	7.59	6.06	−1.53
C16	6.90	12.20	5.30
C17	6.49	10.87	4.38
C18	20.51	5.13	−15.38
C19	9.54	7.91	−1.62
C20	12.24	8.11	−4.14
C21	10.42	11.84	1.43
C22	13.21	14.71	1.50
**Mean** **±** **SD**	**11.08 (5.55)**	**10.41 (4.99)**	**−0.67 (5.09)**
**Minimum**	**0.00**	**1.33**	**−15.38**
**Maximum**	**22.73**	**22.22**	**10.94**

The mean multiple pregnancy delivery rate (MDR) in all 22 IVF Centers was 11.08 ± 5.55% (range 0.00–22.73%) in 2017 and 10.41% ± 4.99 (range 1.33–22.22%), as presented in Table 3.

The cryopreservation incidence (CI) on delivery rate showed a mean of 28.57 ± 19.61% (0–68.25%) in 2017 and a mean of 32.19 ± 20.07% (0–66.34%) in 2018 with a medium increment of 3.62 ± 11.06% between the 2 years analyzed (Table 5).

**Table 5:** Cryopreservation incidence on cumulative delivery RATE % in all 22 IVF centers in the period 2017–2018.

	**2017–2018 Lombardy ART data analysis**		
	**Crypreservation incidence on delivery rate %**		
**ART CENTER**	**2017**	**2018**	**DELTA**
C1	47.50	56.2	8.73
C2	25.13	39.7	14.59
C3	3.36	2.6	−0.78
C4	31.75	50.6	18.86
C5	68.25	66.3	−1.91
C6	55.17	45.6	−9.56
C7	37.50	13.3	−24.17
C8	0.00	0.0	0.00
C9	31.15	36.5	5.33
C10	22.73	19.8	−2.97
C11	35.29	21.4	−13.87
C12	5.88	16.1	10.19
C13	2.70	15.4	12.68
C14	35.58	42.4	6.85
C15	53.42	57.0	3.56
C16	7.41	8.3	0.93
C17	32.95	33.7	0.71
C18	16.13	43.2	27.11
C19	49.88	51.7	1.80
C20	31.78	37.3	5.47
C21	34.88	49.3	14.37
C22	0.00	1.7	1.72
**All (mean)**	**28.57**	**32.19**	**3.62**
**All (SD)**	**19.61**	**20.07**	**11.06**
**ALL (minimum)**	**0.00**	**0.00**	**−24.17**
**All (maximum)**	**68.25**	**66.34**	**27.11**

The delta percentage difference between the 2 years was −0.67 ± 5.09% with a range from −15.38 to + 10.94% (Table 5).

The mean cost for delivery was 26,227 ± 14,737 (range 12,480–76,725) € in 2017 and 25,018 ± 16,039 (range 12,973–86,203) € in 2018 (Table 6). Analyzing the 2 years performance, we find a mean cost reduction of −1,210 ± 6,327 Euros, with a maximum 2018 cost reduction of 14,694 Euros and a maximum 2018 higher cost of 9,478 Euros with data detailed for each center in Table 6. The overall cost was 66,843,000 Euros excluding induction therapy, luteal phase support, pregnancy, delivery and neonatal care costs for 3,348 deliveries and 3,665 and 66,865,000 € for 3,590 live babies born in 2017.

**Table 6:** Cost for the NHS of delivery in all 22 IVF Centers in the period 2017–2018 (conversion Euro to US dollar 1.17).

	**Total cost for delivery (Euros)**		
**ART CENTER**	**2017**	**2018**	**DELTA**
C1	17,838	18,845	1,007
C2	16,067	20,065	3,998
C3	19,101	20,859	1,758
C4	22,211	19,482	−2,728
C5	17,876	15,543	−2,333
C6	16,254	12,973	−3,282
C7	33,385	19,990	−13,395
C8	37,607	45,614	8,006
C9	18,145	16.104	−2.041
C10	15,356	15,356	0
C11	76,725	86,203	9,478
C12	20,301	14,563	−5,739
C13	23,114	23,772	658
C14	17,468	22,370	4,902
C15	20,848	20,091	−757
C16	47,017	32,323	−14,694
C17	27,961	19,487	−8,474
C18	30,404	35,192	4,788
C19	12,480	13,134	654
C20	21,666	26,361	4,696
C21	19,500	15,632	−3,868
C22	45,679	36,430	−9,249
**Mean** **±** **SD**	**26,227 (14,737)**	**25,018 (16,039)**	**−1,210 (6,327)**
**Minimum**	**12,480**	**12,973**	**−14,694**
**Maximum**	**76,725**	**86,203**	**9,478**

## Discussion

In this study, we show fresh cycle DR, CDR, CI, MDR, and cost for delivery of all public funded Lombardy county IVF centers is extremely different among centers in the same region.

To the best of our knowledge, this is one of the first contributions that investigated the impact of CDR in total costs of ART procedures and how different center policies and performances affects this specific variable.

The mean number of pregnancies lost to follow up was lower than 10%, considered in general an acceptable reporting rate level by most ART Registers. However, some centers had a loss to follow up of reported pregnancies as high as 47%, leading to no news about pregnancy outcome and complications as high as nearly 50% of the obtained pregnancies and probably too confusing self-reported pregnancy rates.

The mean DR for fresh embryo transfers as shown in Table 1, had an important 2017–2018 overall mean improvement from 13.23 to 19.33% without a higher 2017–2018 mean multiple pregnancy rate as shown in Tables 1, 2 and although female age is significantly higher in our country than in others and in Lombardy County specifically. Some centers showed significant improvement without a higher multiple rate, but data show an impressive variability among centers not fully justified by any clinical variability in the treated population or by the number of embryos transferred. DR in fresh transfer probably penalize centers where younger more favorable prognosis is always canceled for the risk of hyperstimulation or single embryo transfer is more often performed and benefit centers transferring more than one embryo even in higher risk conditions (17, 18). Fresh transfer performance is a poor and rather confusing (19, 20) than CDR, since frozen embryos are a yearly growing success experience and need probably to be dismissed from all reports. MDR, although a reduction in the 2017–2018 period of data extraction was observed, remained as high as 22% in one IVF center as shown in Table 3.

The last (2016 data) 2020 European Register CDR calculation included data from 38 countries where an overall rate of 29.6% was reported with a range of 4.10–51.80%. The Italian overall rate was in 2016 18.60% (3). Lombardy County 2018 CDR was higher than the Italian overall percentage (21.32 vs. 19.4%) with a 2017 vs. 2018 overall better performance (+1.46%). However, differences in single center CDR ranged from 4.24 to 37.11%. Our data strongly support the conclusion that CDR are extremely different not only among different European countries as reported in 2020 by the European Register, but even among the same region of public funded IVF centers, as shown in 2010 by the 1 year pregnancy rate among all the Netherland's public funded clinics (9). Some Lombardy IVF centers performance was in the higher European ranking (>35% CDR), but others had an extremely low and not improving CDR as shown in Table 2.

The benefit taken from additional FER (over the DR from fresh embryo transfers) was 10.5%, with a highest benefit recorded of 26.1% and the lowest of 0% according to the European Register (3).

Lombardy IVF centers 2018 incidence of FER and FO on CDR was higher than the overall reported by European Register (32.19 vs. the 10.5%) with a benefit as high as 66.34%.

Italian oocyte utilization is different from other countries, since, even after in 2009, the Constitutional Court removed the prohibition of embryo cryopreservation and the limitations to three oocytes only to be utilized, immediately improving overall results (21, 22), all IVF centers decided not to fertilize all the mature oocyte's retrieved, but to cryopreserve oocytes and embryo to reduce the number of stored embryos and the total number of oocytes to be utilized is a decision of each center, even in few cases superior to 12 oocytes. This different policies in oocyte utilization even if FO deliveries are reported, are a bias to make comparison with other countries and made it probably not possible.

We conducted a long analysis to evaluate differences in centers performance influencing the cost for baby born and our conclusions show that the total cost for ART procedure is extremely low in relation of the 2019 total Regional health care budget (0.34%) and the mean cost for delivery was probably acceptable for the country with the lower delivery rate and the higher women mean age at delivery in Europe (23). However, enormous difference was found among centers in our results leading to 12,973 Euros (15,158 US dollars) to 86,203 Euros (100,962 US dollars) cost for delivery.

The risk of multiple pregnancy in centers with a greater propensity to cryopreservation is probably associated with the implementation of more elective single embryo transfers (eSET) which is associated with a multiple pregnancy incidence reduction (24, 25) and a significant progressive decrease in the risks for the mother related to oocyte retrieval (26) and in the costs of drugs needed to induce ovulation, pregnancy care and interventions required for neonatal complications. Considering the higher costs for both gestation and neonatal care in multiple pregnancies, we speculate that the inclusion of these expense items would further acquire the cost differences per live birth and the observed trend and probably cover the total ART procedure costs (5).

Some limitations of our study should be recognized. First, we took some necessary arbitrary and debatable decisions. We cannot exclude that in our setting these variables could have an influence. The economic analysis deserves a critical evaluation. Interventions with an unfavorable economic profile are usually not supported by public health policies, however, we do not believe that our data can yet be used by the public health providers to implement a restriction policy, in relation to couples with poor prognosis. Results from economic analyses are markedly influenced by the basal assumptions and results may differ substantially with the use of different models. Furthermore, a substantial part of IVF associated costs (i.e., pregnancy and neonatal care costs) were not included in the model. Consequently, conclusion applicable to the management of public resources cannot be drawn. The data reported in the present study refer to the 2-year period 2017–2018. These are the most recent data published by the Italian National Center for Diseases, Prevention, and Health Promotion for which the outcomes of all achieved pregnancies are available. We therefore deem that their synthesis could represent the current situation as reliably as possible but, obviously we cannot be sure. Finally, our results are based on the data provided by the 22 Lombardy county public funded IVF Units. This implies that these findings cannot be considered valid for the remaining national and extra-national territory. In central and southern Italy, for example, many patients turn to private centers which, for many reasons, cannot be compared to public ones. Looking at Europe, the average number of public IVF Units per inhabitant is significantly lower than in Lombardy with a consequent different distribution of resources. Furthermore, national health care systems are financed by different models of funding (e.g., tax revenue or multi-payer system and municipal or national healthcare funding). More robust prospective evidence from other contexts is thus warranted.

In conclusion our results highly support in a very different setting data published by Lintsen et al. (9) which demonstrated that differences in 1-year ongoing pregnancy rates between public funded IVF centers exist, even after adjustment for sampling variation and patient mix. Our focus on delivery rate was more detailed in understanding the real costs for baby born, than the cited manuscripts. Unfortunately, our reported data in this publication are retrospective, aggregated data with all the possible bias related to this specific context. We consider our findings as fundamental for the formulation of specific KPI's (Key performance indexes) and minimal performance indexes (PI) (27) as a basis to the allocation of public or insurance resources, leading to a more general spread of ART even in lower resources countries.

## Data Availability Statement

Publicly available datasets were analyzed in this study. This data can be found here: National Institute of Health Italian National Register.

## Ethics Statement

Ethical review and approval was not required for the study on human participants in accordance with the local legislation and institutional requirements. The patients/participants provided their written informed consent to participate in this study.

## Author Contributions

PL-S, GS, and ABu: conception and design. PL-S, ABu, ABo, RD, and GS: data extraction, interpretation, and critical revision of the manuscript. RD: analysis. PL-S: drafting the manuscript. All authors contributed to the article and approved the submitted version.

## Conflict of Interest

The authors declare that the research was conducted in the absence of any commercial or financial relationships that could be construed as a potential conflict of interest.

## Publisher's Note

All claims expressed in this article are solely those of the authors and do not necessarily represent those of their affiliated organizations, or those of the publisher, the editors and the reviewers. Any product that may be evaluated in this article, or claim that may be made by its manufacturer, is not guaranteed or endorsed by the publisher.
